# Do Women have Adequate Knowledge about Pelvic Floor Dysfunctions? A Systematic Review

**DOI:** 10.1055/s-0039-1695002

**Published:** 2019-08

**Authors:** Júlia Ferreira Fante, Thais Daniel Silva, Elaine Cristine Lemes Mateus-Vasconcelos, Cristine Homsi Jorge Ferreira, Luiz Gustavo Oliveira Brito

**Affiliations:** 1 Faculdade de Ciências Médicas, Universidade Estadual de Campinas, Campinas, SP, Brazil; 2 Faculdade de Medicina, Universidade de São Paulo, Ribeirão Preto, SP, Brazil

**Keywords:** knowledge, pelvic floor, urinary incontinence, systematic review, conhecimento, assoalho pélvico, incontinência urinária, revisão sistemática

## Abstract

**Objective** We sought to investigate whether women present adequate knowledge of the main pelvic floor disorders (PFDs) (urinary incontinence – UI, fecal incontinence – FI, and pelvic organ prolapse – POP).

**Data sources** A systematic review was performed in the MEDLINE, PEDro, CENTRAL, and Cochrane databases for publications from inception to April 2018.

**Selection of studies** A total of 3,125 studies were reviewed. Meta-analysis was not possible due to the heterogeneity of primary outcomes and the diversity of instruments for measuring knowledge. The quality of the articles included in the analysis was evaluated with the Newcastle-Ottawa Scale (NOS) adapted for cross-sectional studies.

**Data collection** Two authors performed data extraction into a standardized spreadsheet.

**Data synthesis** Nineteen studies were included, comprising 11,512 women. About the methodological quality (NOS), most of the studies (n = 11) presented a total score of 6 out of 10. Validated questionnaires and designed pilot-tested forms were the most frequently used ways of assessing knowledge. Some studies were stratified by race, age, or group minorities. The most used questionnaire was the prolapse and incontinence knowledge questionnaire (PIKQ) (n = 5). Knowledge and/or awareness regarding PFD was low to moderate among the studies. Urinary incontinence was the most prevalent PFD investigated, and the most important risk factors associated with the lack of knowledge of the pelvic floor were: African-American ethnicity (n = 3), low educational level (n = 4), low access to information (n = 5) and socioeconomic status (n = 3).

**Conclusion** Most women have a gap in the knowledge of pelvic floor muscle dysfunctions, do not understand their treatment options, and are not able to identify risk factors for these disorders.

## Abstract (pt)

**Resumo:**

**Objetivos** Nós investigamos se as mulheres possuem adequado nível de conhecimento sobre as principais disfunções do assoalho pélvico (incontinência urinária – IU, incontinência fecal – IF, e prolapso de órgãos pélvicos – POP).

**Fontes de dados** Uma revisão sistemática foi realizada nas bases de dados MEDLINE, PEDro, CENTRAL e Cochrane com publicações até abril de 2018.

**Seleção dos estudos** Foram triados 3.125 estudos. A metanálise não foi possível devido a heterogeneidade dos desfechos analisados e a diversidade de instrumentos para aferir o conhecimento. A qualidade dos artigos incluídos na análise foi avaliada pela escala de Newcastle-Ottawa (NOS) adaptada para estudos transversais.

**Extração de dados** Dois autores fizeram a extração em uma planilha previamente testada.

**Síntese de dados** Dezenove estudos foram incluídos, totalizando 11.512 mulheres. A NOS apresentou um score de 6 (total = 10) na maioria dos estudos (n = 11). Para a avaliação do conhecimento do assoalho pélvico, questionários validados e testados de forma piloto foram empregados. Alguns estudos foram estratificados segundo raça, idade, ou minorias. Encontrou-se baixo a moderado nível de conhecimento e/ou percepção sobre as disfunções do assoalho pélvico. O mais usado foi o *prolapse and incontinence knowledge questionnaire* (PIKQ) (n = 5). A IU foi a disfunção pélvica mais investigada, e os fatores de risco mais importantes associados com a falta de conhecimento foram: etnicidade afro-americana (n = 3), nível baixo educacional (n = 5), baixo acesso a informação (n = 5), e status socioeconômico (n = 3).

**Conclusão** A maioria das mulheres leigas tem uma lacuna de conhecimento sobre as disfunções do assoalho pélvico, baixo conhecimento sobre opções de tratamento e sobre os fatores de risco para essas disfunções.

## Introduction

Pelvic floor muscle (PFM) dysfunctions have a negative impact in the quality of life of many women. These dysfunctions mainly include pelvic organ prolapse (POP), urinary incontinence (UI), and fecal incontinence (FI).^1^ Female stress urinary incontinence and pelvic organ prolapse (POP) are prevalent conditions and are rarely associated with severe comorbidities, despite the costs and restriction caused to women's lives.^2^ The prevalence of POP varies from 2 (symptomatic women) to 50% o(women with clinically insignificant POP).^3^ Meanwhile, the prevalence of UI reaches indices varying between 10 and 58% in women living at community settings and 50 to 84% in women residing at long-permanence institutions.^4^ Annual health costs related to UI care in the USA exceed 16 billion dollars. Despite the prevalence and the cost for treating PFM dysfunctions, many women do not receive adequate attention. Less than 50% of incontinent women seek for medical treatment.^2^ Pelvic floor muscle treatment (PFMT), bladder training, and other conservative approaches are considered the first line of treatment for women who suffer PFM dysfunctions. However, many of these women do not have information or knowledge regarding conservative treatment for PFM disorders.^5^ There are studies that have addressed the knowledge of patients regarding these dysfunctions, but with no compiled data on this matter. This increases the chances of successful therapy, changes in life habits, and reductions on disease's symptoma.^2^ Thus, our study aimed to perform a systematic review of women's knowledge about the pelvic floor structures (muscles, ligaments, organs), its functions, dysfunctions, and possible conservative treatments for each disorder by measurement through surveys, questionnaires, or any available instrument within the literature.

## Methods

### Eligibility Criteria and Study Selection

The eligibility criteria were scientific articles and juts cross-sectional studies (cross-sectional scientifics articles) in English language that investigated women's knowledge regarding the pelvic floor (PF) functions and/or dysfunctions and possible conservative treatments for them. Studies that aimed to focus on health professionals or that were not specifically aiming to understand women's knowledge of the pelvic floor, studies involving pregnant and postpartum patients, those with qualitative designs, or quantitative studies that did not separate data according to gender were excluded from the analysis.

### Information Sources and Search

The last literature search was performed on April 2018 and included studies from inception. The consulted databases were: Medline/PubMed, PEDro, Cochrane Central Register of Controlled Trials and Cochrane Database of Systematic Reviews. The overall search strategy used was (*knowledge* OR *comprehension* OR *education* OR *education level*) (*urinary incontinence* OR *pelvic organ prolapse* OR *genital prolapse* OR *stress urinary incontinence* OR *urgency urinary incontinence* OR *mixed urinary incontinence* OR *cystocele* OR *rectocele* OR *apical prolapse* OR *uterine prolapse* OR *overactive bladder* OR *detrusor overactivity*) NOT (*m?n* OR animal*). A detailed example of search strategy (Pubmed) is illustrated in Appendix 1.

### Screening and Data Extraction

Data search was performed by authors (J. F. F. and T. D. S.), and if a study was not a common decision to include or exclude, a third author (L. G. O. B.) was included to come to a consensus. A standardized data extraction form was used to collect the following data: authors, year of publication, journal, country of origin, sample, age (years), objectives, outcome measure, and results/conclusions. Data extraction was performed by two independent raters (J. F. F. and T. D. S.).

### Outcomes

The primary outcome was knowledge regarding the pelvic floor muscles, ligaments or organs, and related disorders, measured by a questionnaire that could be previously prepared (e.g. incontinence quiz, prolapse and incontinence knowledge questionnaire) or prepared by the authors (previously or not pilot-tested). Knowledge could also be assessed with or without attitude and/or practice (Knowledge, attitude, and practice - KAP) format. Answers for knowledge could be categorical or as continuous variable (e.g. score results).

### Risk of Bias Assessment and Quantitative Analysis

Assessment of methodological quality was performed by the Newcastle-Ottawa Scale adapted for cross-sectional studies. This scale was originally developed to assess the quality of observational studies and contains eight items that assesses three domains: selection, comparability and outcome. The score was divided into: good quality (3–5 stars in selection, 1–2 stars in comparability, 2–3 outcome), fair quality (2 stars in selection, 1–2 in comparability and 2–3 in outcome) and poor quality (0–1 star in selection, 0 star in comparability and 0–1 star in outcome).^6^ ^7^

As data were extracted and described, heterogeneity between the outcomes did not reach possibility for pooling data and performing subgroup analysis or metanalysis. Results were displayed in tables in a synthesized format.

## Results

### Characteristics of the Selected Studies and Newcastle-Ottawa Scale Quality Assessment

**Fig. 1:**
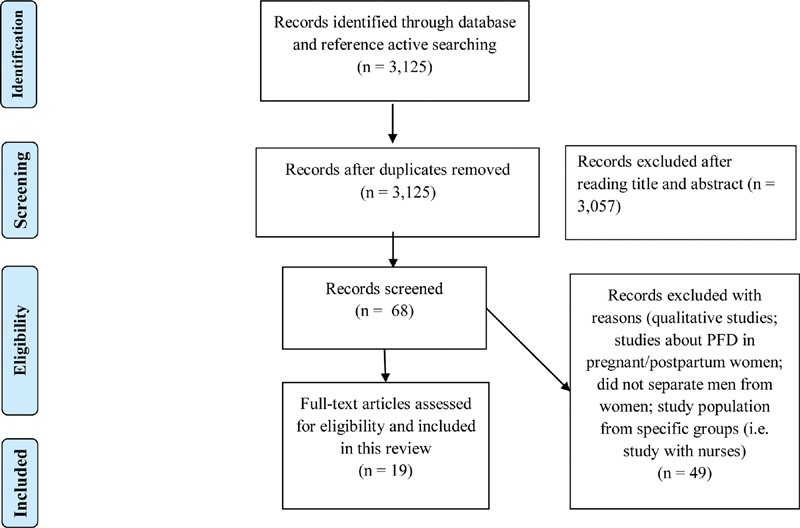
PRISMA flow diagram for the selected studies. Abbreviations: PRISMA, preferred reporting items for systematic reviews and meta-analyses; PFD, pelvic floor disorders.

Figure 1 shows all the pathways for this systematic review. Database searches identified a total of 3,125 studies with no duplicates, and after excluding title and abstract, 68 studies remained for screening. The reasons for exclusion are explained in the flowchart. Despite having found 19 articles for data extraction, some aspects of these studies were highly heterogeneous, such as sampling, methods of investigating the subjects' knowledge, and knowledge as primary outcome.

Almost all studies were cross-sectional,^8^ ^9^ ^10^ ^11^ ^12^ ^13^ ^14^ ^15^ ^16^ ^17^ ^18^ ^19^ ^20^ ^21^ ^22^ ^23^ ^24^ ^25^ except for one pilot study^26^ that included a systematic review. Most of the studies were from United States (n = 8) and regarding the time period of the studies, 7 studies did not inform the time period of data collection,^11^ ^13^ ^16^ ^19^ ^20^ ^25^ ^26^ and the others informed different durations (from 3 weeks to 21 months).^8^ ^9^ ^10^ ^12^ ^14^ ^15^ ^17^ ^18^ ^21^ ^22^ ^23^ ^24^ From these retrieved studies, a total of 11,512 women were included, with a mean age varying from 17 to 77 years (Table 1). Some studies focused only on UI,^10^ ^15^ ^19^ ^22^ ^25^ ^26^ while other studies investigated other pelvic floor disorders (PFDs), pelvic floor function (PFF) or pelvic floor symptom (PFS),^8^ ^9^ ^11^ ^12^ ^14^ such as POP.^18^ Regarding the NOS quality assessment, most of the studies scored 6 (n = 11) on a scale varying from 0–10. The maximum score obtained by the studies in the present review was 7 (n = 4), 3 studies were classified with score 5 (fair quality), and only 1 article with a score of 3 (Table 2).

**Table 1:** General characteristics of the selected studies

References	Study Design/Period	Country	Sample (n)	Mean age ± SD (range)	Objectives
Freitas et al (2018)^8^	CS/From January 2016 to October 2017	Brazil	133	53.3 (13.8)	To assess the level of knowledge about PFMs and the relationships between PFM knowledge and the ability to contract the PFMs, PFM strength, and prevalence of UI.
Arbuckle et al (2018)^9^	CS/From August 2015 to June 2016	United States of America	216	17 ± 2.1 (14–17; 18–21)	To determine the prevalence and awareness of pelvic floor disorder (PFD) symptoms among adolescent females. Patient awareness of these disorders and awareness of pregnancy as a risk factor for PFD were also investigated
Cardoso et al (2018)^10^	CS/March–June 2016	Brazil	118	21.6 ± 2.7	To evaluate the prevalence of UI in female athletes practicing high-impact sports and its association with knowledge, attitude, and practice (KAP).
Neels et al (2016)^11^	CS/NI	Belgium	212	21.6 (18–27)	To evaluate the knowledge of PFF in young nulliparous women.
Parden et al (2016)^12^	CS/2014–2015	United States of America	1092	23.5 ± 3.1 (19–30)	To characterize lower urinary tract and PFS prevalence, awareness of these symptoms in women in general and in their family members
Dunivan et al (2015)^13^	CS/NI	Mexico	144	77.7 ± 9.1 (55–90)	To evaluate knowledge about UI and POP among elder southwestern American-Indian women and to assess knowledge by comparing questionnaire scores of these American-Indian women to historic controls.
Mandimika et al. (2015)^14^	CS/Febr2010–Aug2011	United States of America	416	52.5 ± 18.0; 38.3 ± 15.2; 50.2 ± 17.5 (19–98)	To compare PFD knowledge among women of different racial/ethnic groups, focusing on aspects of knowledge that are more likely to influence patient behavior, including PFD risk factors and treatment options.
Day et al (2014)^26^	PS/NI	Ireland	36	NI – included 50+	To describe community-dwelling Irish women's knowledge of UI.
Perera et al (2014)^15^	CS/3 weeks	Sri Lanka	400	41.94 (21–88)	To determine the prevalence, degree of severity, identify associated factors and study the perceptions and health seeking behavior of women with SUI attending a health care facility.
Shrestha et al (2014)^16^	CS/NI	Nepal	4,693	30.0 ± 7.4	To assess UP knowledge among married women of reproductive age and to determine the association between UP knowledge and socioeconomic characteristics.
Mandimika et al. (2014)^17^	CS/ Feb 2010–Aug 2011	United States of America	431	49.2 ± 17.9 (19–98)	To investigate baseline knowledge and demographic factors associated with a lack of knowledge about UI and POP.
Good et al (2013)^18^	CS/ May2011–Aug 2012	United States of America	213	58.9 ± 14.1	To describe patient knowledge about POP diagnosis and treatment, and patient attitudes regarding the uterus in women seeking care for POP symptoms.
Morhason-Bello et al (2012)^19^	CS/NI	Nigeria	1,955	34.8 (15–65)	To describe the perceived causes of UI and factors associated with awareness of causes of UI among women in the community.
Kang (2009)^20^	CS/NI	Korea	182	51.2	To explore knowledge and attitudes about UI among Korean-American women with incontinence and provide initial information.
Shah et al (2008)^21^	CS/ March–December 2006	United States of America	126	35.7	To assess the knowledge of UI and POP of white women versus non-white women.
Wong et al (2006)^22^	CS/3 months	China	540	(17–77)	To assess the prevalence, knowledge, and behavior of the search for treatment of UI by Chinese women.
Kubik et al (2004)^24^	CS/May2002–Febr2003	United States of America	212	(35–80)	To investigate if UI knowledge is different between white and minority groups, and if there is an association between SES and UI knowledge, and if SES explains differences in UI knowledge between white and minority race/ethnic groups.
Kim et al (2004)^23^	CS/ Dec2002–Jan2003	Korea	276	(55–97)	To examine the prevalence of UI and UI-related knowledge among community-dwelling Korean women aged 55 and over.
Kubik et al (2004)^24^	CS/May2002–Febr2003	United States of America	212	(35–80)	To investigate if UI knowledge is different between white and minority groups, if there is an association between SES and UI knowledge, and if SES explains differences in UI knowledge between white and minority race/ethnic groups.

Abbreviations: BT, bladder training; CS, cross-sectional; FI, fecal incontinence; NI, not informed; PF, pelvic floor; PFDs, pelvic floor disorders; PFEs, pelvic floor exercises; PFF, pelvic floor function; PFMT, pelvic floor muscle training; PFM, pelvic floor muscle; PFS, pelvic floor symptoms; PS, pilot study; POP, pelvic organ prolapse; SES, socioeconomic status; SUI, stress urinary incontinence; UI, urinary incontinence; UP, uterine prolapsed; KAP, knowledge, attitude, and practice.

**Table 2:** Methodological quality (as per Newcastle-Ottawa scale), independent variable(s), instruments, and main results of selected studies

References	Quality	Independent Variables	Questionnaires	Results
Freitas et al (2018)^8^	Selection: ✸✸✸ Comparability: ✸ Outcome: ✸✸	Knowledge/ PFM	Designed questionnaire but no information if pilot-tested; ICIQ-UI-SF	Most of the women presented no PFM knowledge, with a mean total score of 0.48 (±0.97). The ICIQ-UISF mean score was 7.1 (±6.8). There were weak correlations between PFM knowledge and age (r −0.2044/ p = 0.01), and PFM knowledge and parity (r −0.19568/*p* = 0.02). Pelvic floor muscle knowledge was higher among women with higher education levels (*p* = 0.0012) and those who had previously performed PFM training (*p* < 0.001).
Arbuckle et al (2018)^9^	Selection: ✸ Comparability: ✸ Outcome: ✸	Awareness; PFD	ISI-P; FISI; ISI-2; POPDI-6; Designed questionnaire but no information if pilot-tested	The majority of respondents had at least heard about UI and FI (62.9%). The prevalence of any UI was 31.5 %. Approximately 29% of adolescents reported an interest in learning more about pelvic floor disorders. Early education regarding PFS may lead to prevention or empowerment to seek treatment as adolescents age.
Cardoso et al (2018)^10^	Selection: ✸✸✸✸ Comparability: ✸ Outcome: ✸✸	IU; KAP	KAP; ICIQ-SF; QOL; Designed and pilot-tested questionnaire	The prevalence of UI in 118 athletes was 70% (82). Regarding the KAP survey, 31% of the athletes (37) demonstrated adequate knowledge, 53% (63) adequate attitude, and zero adequate practice. Athletes with adequate knowledge were 57% less likely to develop UI.
Neels et al (2016)^11^	Selection: ✸✸✸ Comparability: ✸ Outcome: ✸	Knowledge of PFF	Designed and pilot-tested questionnaire	Using a VAS scale (0–10), the women rated their knowledge about the pelvic floor as a mean of 2.4 (SD 2.01). A total of 93% of the women were insufficiently informed and requested more information; 25% had concerns about developing UI, and 14% about FI. Many of the women were unaware what pelvic floor training meant.
Parden et al (2016)^12^	Selection: ✸✸✸ Comparability: ✸ Outcome: ✸✸	PFS (UI, POP, FI, PFS's knowledge)	Designed questionnaire but no information if pilot-tested	There was no difference between groups in awareness of family members with UI, FI, or POP symptoms (*p* ≥ 0.24). Young women were more likely to have received education regarding UI; (aOR 2.6, 95% CI 1.8–3.6), FI (aOR, 3.3; 95% CI, 2.2–4.8), POP (aOR, 2.9; 95% CI, 2.1–4.2), and have greater understanding regarding the causes of UI (aOR, 2.9; 95% CI, 1.7–4.8), FI (aOR, 1.6; 95% CI, 1.1–2.3), and POP (aOR, 1.9; 95% CI, 1.3–2.9).
Dunivan et al (2015)^13^	Selection: ✸✸✸ Comparability: ✸ Outcome: ✸	UI, POP's Knowledge	PIKQ; BICS-Q	The mean (SD) for PIKQ of UI score was 6.6 (3.0) (similar to historic gynecology controls 6.8 [3.3], *p* = 0.49), and the mean (SD) for PIKQ on POP score was 5.4 (2.9) (better than historic gynecology controls 3.6 [3.2], *p* < 0.01).
Mandimika et al (2015)^14^	Selection: ✸✸✸ Comparability: ✸ Outcome: ✸✸✸	PFD's Knowledge stratified by race	PIKQ	African-American women presented higher odds for lack of knowledge in UI and POP etiology (aOR 3.05 95%CI [1.70–5.47] and aOR 2.15 95%CI [1.18–3.91], respectively) but no difference with regard to UI and POP diagnosis.
Day et al (2014)^26^	Selection: ✸✸ Comparability: ✸ Outcome: ✸✸	UI's Knowledge	UIK	Participants had poor knowledge of UI, especially in relation to risk, prevention, treatment, and management factors. Less than 20% of the participants indicated they had been given information on bladder and bowel health issues
Perera et al (2014)^15^	Selection: ✸✸✸ Comparability: ✸ Outcome: ✸✸	SUI; Perceptions and Health seeking behavior	Designed and pilot-tested questionnaire	Stress urinary incontinence was perceived as an illness by 210 (52.5%) subjects. Stress urinary incontinence was significantly associated with pregnancy, parity, vaginal delivery, complicated labor, diabetes mellitus, chronic cough, constipation, and fecal incontinence (*p* < 0.05).
Shrestha et al (2014)^16^	Selection: ✸✸✸ Comparability: ✸ Outcome: ✸✸	UP's Knowledge	Designed and no information if pilot-tested	Fifty-three percent had never heard about UP. Among women who had heard about UP, 37.5% had satisfactory knowledge. Any knowledge about UP was associated with both urban and rural settings, age group, and education level. However, satisfactory knowledge about UP was associated with administrative region, ecological zones, caste/ethnic group, and age group of women
Mandimika et al (2014)^17^	Selection: ✸✸✸ Comparability: ✸ Outcome: ✸✸	UI; POP	PIKQ	There is a global lack of knowledge about UI and POP among community-dwelling women, with more pronounced knowledge gaps among nonwhite women.
Good et al (2013)^18^	Selection: ✸✸ Comparability: ✸ Outcome: ✸✸✸	POP's Knowledge	Designed and no information if pilot-tested	Prolapse-related knowledge is low in women seeking care for prolapse symptoms.
Morhason-Bello et al (2012)^19^	Selection: ✸✸✸ Comparability: ✸ Outcome: ✸✸	UI	Designed and no information if pilot-tested	There was a lower odd of awareness of the UI etiology among women less than 30 years, with lower level of education, from rural areas, with five or more children and without history of urine leakage.
Kang (2009)^20^	Selection: ✸✸✸ Comparability: ✸ Outcome: ✸✸	IU; Knowledge	Incontinence quiz	Results suggest that Korean-American women are less knowledgeable and have more negative attitudes toward UI than the general population.
Shah et al (2008)^21^	Selection: ✸✸✸ Comparability: ✸ Outcome: ✸✸	UI; POP	PIKQ	Punctuation mean for white women was higher than non-white at UI scale (*p* =.019), but not at POP scale (*p* =.354). Regardless of race, both groups had a higher knowledge for UI than for POP.
Wong et al (2006)^22^	Selection: ✸✸✸✸ Comparability: ✸ Outcome: ✸✸	UI	UDI-6/ IIQ-7	A total of 78.3% of the interviewed women did not know that UI is a disease, and 60.6% of them thought urine loss is a normal part of the aging process.
Kim et al (2004)^23^	Selection: ✸✸✸✸ Comparability: ✸ Outcome: ✸	UI's Knowledge	Questions derived from BFLUTSQ and Incontinence Quiz	More than 50% of respondents incorrectly agreed that “UI is the result of normal aging”. Only 20.9% knew that there were exercises that control urine leakage after strain. Older women who had sought treatment had higher mean score for UI-related knowledge.
Kubik et al (2004)^24^	Selection: ✸✸✸✸ Comparability: ✸ Outcome: ✸✸	UI's Knowledge; SES	Incontinence Quiz	White women scored better than minority women on the incontinence quiz. Socioeconomic status explains racial differences in total UI knowledge.
Keller (1999)^25^	Selection: ✸✸✸✸ Comparability: ✸ Outcome: ✸	UI's Knowledge	Incontinence Quiz	Over half of the 117 respondents incorrectly indicated that incontinence is a normal result of advanced age, almost one third of the respondents incorrectly believed that most people become incontinent by the time they reach the age of 85.

**Abbreviations:** BICS-Q, barriers to incontinence care seeking questionnaire; BT, bladder training; CI, confidence interval; FI, fecal incontinence; IIQ-7, incontinence impact questionnaire short form; ICIQ-UI-SF, international consultation on incontinence questionnaire on urinary incontinence-short form; PF, pelvic floor; PFDs, pelvic floor disorders; PFC, pelvic floor complications; PFEs, pelvic floor exercises; PFF, pelvic floor function; PFS, pelvic floor symptom; PFMT, pelvic floor muscle training; PS, pilot study; PIKQ, prolapse and incontinence knowledge questionnaire; POP, pelvic organ prolapse; SD, standard deviation; SUI, stress urinary incontinence; SES, socioeconomic status; UI, urinary incontinence; UIK, urinary incontinence knowledge scale; UDI-6, urogenital distress inventory short form; UP, uterine prolapse; VAS, visual analogue scale; KAP, knowledge, attitude and practice.

There were risk factors that were mostly related to the lack of knowledge of pelvic floor (PF) such as educational level, access to information, socioeconomic status, age and race (Table 3).

**Table 3:** Most frequently cited risk factors for lack of knowledge of pelvic floor dysfunction

Variables	References
**African-American ethnicity**	Mandimika et al (2014, 2015),^14^ ^17^ and Shah et al (2008)^21^
**Low educational level**	Mandimika et al (2014),^17^ Good et al (2013),^18^ and Morhason-Bello et al (2012)^19^
**Low access to information**	Neels et al (2016),^11^ Parden et al (2016),^12^ and Dunivan et al (2015)^13^
**Low socioeconomic status**	Shrestha et al (2014),^16^ Morhason-Bello et al (2012),^19^ and Kubik et al (2004)^24^

### Questionnaires

All selected studies have utilized validated^8^ ^9^ ^10^ ^13^ ^14^ ^17^ ^21^ ^22^ ^23^ ^24^ ^25^ ^26^ questionnaires, developed or not by the authors.^8^ ^9^ ^10^ ^11^ ^12^ ^15^ ^16^ ^18^ ^19^ ^23^ Some developed questionnaires were validated by the authors.^10^ ^11^ ^15^ Nells et al^11^ have validated the questionnaire with experts and non-trained volunteers, and both groups presented low interobserver variability. Perera et al^15^ validated their questionnaire by a pretest that analyzed the questionnaire content, and Cardoso et al^10^ have utilized the assessment of seven experts in the gynecology/ womens' health area by a Delphi panel to analyze the concept and relevance of the elaborated questions. The most cited questionnaires were: prolapse incontinence knowledge questionnaire (PIKQ) (n = 5), incontinence quiz (n = 4), urinary incontinence knowledge (UIK), urogenital distress inventory short form (UDI-6) and incontinence impact questionnaire short form (IIQ-7) (n = 1); Bristol female lower urinary tract symptom (BFLUTS) and UI-related questionnaire (n = 1).

### Knowledge about Pelvic Floor Anatomy and Function

Four studies were included,^8^ ^9^ ^11^ ^12^ one^11^ assessed the knowledge of nulliparous women regarding the pelvic floor functions. It was found that women presented some knowledge regarding some functions of the pelvic floor, such as pelvic floor structure and function, since 93% of women knew about the existence of muscles in this region, and 92% managed to locate this region. However, few of them had knowledge about the role of pelvic floor anatomy on sexual function (6.2–64.3%). Furthermore, most of them did not know how many openings exist in the female pelvic floor. It was concluded that most of the patients (81%) had never received information regarding the pelvic floor.

Arbuckle et al^9^ analyzed the prevalence and the knowledge of PFD in adolescents (14–21 years). They have observed that the prevalence of UI was 31.5%, and urge incontinence was present in 15.7% of the women included in the study. Regarding knowledge, only 19.5% and 5.1% of the participants had heard about POP and FI, respectively. Furthermore, discussion about PFD at schools was also low (1.9–6.5%) within this group, and 29.4% of the adolescents would want to know more about the topic. Parden et al^12^ have shown that even with low rates of symptoms in both groups, the adolescent women (19–24 years) and young women (25–30 years) groups had similar interest in learning more about PFD (33.9% vs 31.4%, *p* = 0.45). After stratifying the groups by age and educational level, it was found that, when compared to adolescents, female young adults were more prone to receive education regarding UI, FI, and POP. The same association was found for the group of women with higher education, who had significantly higher rates of willingness to receiving information (UI = 31.5% vs 8.4%, *p* ≤ 0.0001; FI = 24% vs 5.4%, *p* ≤ 0.0001; POP = 27.6% vs 8.2%, *p* ≤ 0.0001) while teenagers were not aware of most of pelvic dysfunctions.

Freitas et al^8^ have analyzed the knowledge of Brazilian women about PFM and its relationship with the capacity to contract the PFM. Most of women (55%) presented a low level of knowledge, and 79.7% did not know the PFM functions. Moreover, a low correlation between PFM knowledge and age was found (*p* = 0.01), and there was a statistically significant difference between the years of education and previous practice of PFMT.

### Knowledge about UI

Eight studies^10^ ^15^ ^19^ ^20^ ^22^ ^23^ ^25^ ^26^ aimed to investigate women's knowledge about UI, and all of them have shown that women had a low knowledge about UI. Most of the studies have also shown that treatment for UI and associated risk factors for UI were not fully understood by the patients, regardless of age and country of origin. Women perceived some risk factors for UI. Day et al^26^ and Keller^25^ have found that women described aging as an important factor for UI. Regarding treatment, women did not look for treatment, and the following reasons were pointed out: lack of knowledge, embarrassment, and UI seen as a minor health issue. These findings were similar to another study performed by Cardoso et al,^10^ in which knowledge, attitude, and practice regarding UI was investigated in high impact athletes. Despite 70% of them have complained about UI during exercise, 96% did not consider this as a problem worthy of seeking help, and none had ever told her coach about the UI.

### Knowledge about POP

One study has only focused on POP.^18^ Good et al^18^ have found that American women presented a lack of knowledge regarding POP, with 44% of them scoring the questionnaire about this subject. Another study^16^ has focused only on uterine prolapse (UP). Shrestha et al^16^ have observed knowledge about UP on married women at reproductive age. Half of them have never heard about UP, and within the group that presented some knowledge about UP, only 37.5% presented a satisfactory level. Women that were living in an urban area presented more chance to have knowledge about UP, as well as higher educational level.

### Knowledge about UI and POP

Two studies^13^ ^17^ have analyzed UI and POP within their objectives, one of which has compared its results with those of control groups. Dunivan et al^13^ used a control group formed by women with PFDs, because they assumed they would have better knowledge if informed during consultations, and compared with healthy women and elderly American-Indian women. The former group presented a higher knowledge score when compared to the other groups. Mandimika et al^17^ found that approximately one third (32.2%) of the participants reported having a history of UI; however, only 4.6% of all women reported being treated for this condition; Also, 6% of the women reported having a problem with POP, but only 4.0% of them reported having been treated by POP. Moreover, 71.2% of the subjects lacked UI proficiency (< 80% was correct), whereas 48.1% lacked proficiency in POP knowledge (< 50% was correct). Regarding the association of risk factors with UI or POP, educational level was the only factor associated with knowledge about UI.

### Knowledge about UI and POP According to Race

Some studies have related the l of treatment seeking for pelvic floor dysfunctions to minority groups. Three studies^14^ ^21^ ^24^ assessed the knowledge separating the subjects by racial groups. Mandimika et al^14^ found that African-American women were more prone to not having adequate knowledge about UI and the etiology and treatment of POP. Furthermore, women did not know that PFMs could be useful for treating UI. Shah et al^21^ identified a higher knowledge level for white women when compared with Asian, Hispanic, and African-American women. Kubik et al^24^ perceived that white women presented a higher score on the incontinence quiz questionnaire compared with other racial groups (6.16 ± 2.86 vs 5.46 ± 2.66, *p* = 0.71) (Hispanic, African-American). Furthermore, higher socioeconomic status (SES) was associated with higher incontinence quiz total score.

## Discussion

This systematic review showed that women's knowledge of PFDs was very limited, and that it could be influenced by socioeconomic variables, such as racial groups. All included studies were quantitative, but this evidence was also found on qualitative studies. Anger et al^27^ performed a focus group of women with overactive bladder to better understand the experiences and level of understanding related to the problem. As a result, it was found that women had no understanding of the cause of overactive bladder, chronicity, and the rationale for various diagnostic tests.

Women's beliefs may also give them a chance to reflect about the cause of their disease. Melville et al^28^ have found that 50% of women suggested an inherent problem with the pelvic floor or bladder as a cause for their symptoms. Obviously, knowledge is connected to the educational and socioeconomic level; thus, cultural aspects are not only the main factor influencing beliefs.

Race is a variable with a possible effect modification. Another point for discussion is that the percentage of surgeries performed for PFDs may be different among racial groups, and this may influence the prevalence of PFDs. If we know that PFDs may differ among racial groups, it will be possible to promote aims focusing on education for this population.^14^ Further cohort studies are necessary to understand this variable as we know that cross-sectional studies cannot establish the route of causality between one variable and the outcome.

Only half or less of women with UI discusses their condition with a health professional.^29^ Even when health professionals are consulted, there are surprisingly low rates of treatment of women with symptoms of UI.^30^ In studies that investigate the reasons why women do not seek treatment for UI, several other themes were identified: shame, belief that incontinence is part of the normal aging process, sensation that they can handle the problem on their own, and low expectations of benefits with treatment.^31^ ^32^ ^33^ ^34^ This information is related with the findings of this review, since the studies that focus on the lack of knowledge have identified the lack of search for treatment due to lack of knowledge, embarrassment, and because some women have considered UI as a small problem and a “normal” part of the aging process.

Jácome et al^35^ observed a high prevalence (30.2–35.8%) of UI in athletes; however, more than half (61.4%) of the athletes had never talked to anyone about their leakage, and 9 (20.4%) reported having discussed the problem with a friend. And when urine loss occurred, the athletes felt concerned, annoyed, frustrated, and fearful that a new activity might trigger another leakage but with no current impact on their daily lives.

It is important to highlight that patients with chronic diseases, such as overactive bladder and UI, seek different information from patients with acute illnesses, regarding diagnosis and treatments available. Furthermore, a study of patients with heart failure found that patients with good disease control have achieved better functional status, suffer less anxiety, and present fewer reports of depression and better quality of life than patients with low perceived control of disease.^36^

Liao et al^37^ administered an educational 4-hour program with pelvic muscle training to a cohort of 55 women with UI in Taiwan. The researchers applied a knowledge questionnaire containing 20 statements of yes/no questions as well as an index of severity of UI and self-perceived severity of UI to patients before and 8 weeks after the educational intervention. The participants showed significant improvement of knowledge scores and reported a significant decrease in the severity of UI.

In a study conducted over a decade ago, Branch et al^38^ found substantial gaps in knowledge about UI among community-dwelling individuals aged 65 years and concluded that levels of knowledge about UI should be increased to ensure that proper treatment and management are achieved. The lack of knowledge about the pelvic floor in women demonstrates the necessity of creating educational programs for health professionals on this topic.

Stadnicka et al^39^ aimed to perform a prophylactic program for Stress Urinary Incontinence (SUI). Through literature review and results of their own investigations, it is concluded that a program for prevention of SUI should include mainly: [1] preparation of health professionals to spread health education among women in the prevention of SUI; [2] the preparation of appropriate educational materials in the form of brochures, leaflets, posters of information on symptoms, causes and prevention of UI indicates that health care available to all women when the disease is suspected or institutions already present, [3] the spread problems related to SUI in the means of mass communication that provide information to a wide audience in order to raise awareness about the significance of this social problem and also in order to break the stereotype associated with this disease, [4] clarifying about the importance of performing exercises for the PFM during pregnancy, and menopause to maintain its own function, and [5] focus on the possibilities of changes in factors that predispose SUI in order to reduce or eliminate these factors.

According to Herbruck,^40^ the costs of UI are financially and socially significant to those who are living with its effects. The determination of possible modifiable factors that cause changes in the UI and in the PF is complicated. A reasonable starting point could be counseling patients about the importance of education and awareness of the PF to improve their quality of life. In addition, health professionals in general should get closely involved to the theme in order to provide quality information that improves with reverse in preventive and rehabilitative care female UI. These data confirm the findings of Kang,^20^ that suggest that the absence of a sharing decision-making process may contribute for an inadequate interpretation of patient symptoms.

The limitations of this review are, mainly, the heterogeneity of measuring knowledge, the non-stratification of baseline sociodemographic variables, such as education level, and the response bias that is implicit to any study that assesses knowledge; maybe these percentages are worse than the findings from each study. It should be highlighted that the research on the PF knowledge had a specific validated questionnaire, and that the interviews between professional and patients were more objective; thus, future studies could reproduce them.

The knowledge about PFM is important for women to know their own bodies, easing comprehension about their orientations and proposed treatment by health professionals. Communication and information are essential for the treatment of patients with PFDs. Correct information is important to obtain consent from patients about proposed therapy during treatment, the increase of participation, reduction of anxiety, increase of knowledge about the disease, and the satisfaction of the patients with the obtained results, which might increase the chances of therapeutic success. This knowledge about the PF showed to be increased through several programs, such as PFMT, behavioral modification, and educational workshops by physicians, physiotherapists and/or nurses.

According to this review, there is a lack of data on the knowledge of adult women regarding to the physiological role of the PF and the ability to contract the PFM. It is important that women receive information on the PFM function and dysfunction. It is also essential to establish models of preventive and rehabilitation activities to be included in women's care in all health care levels.

## Conclusion

Knowledge of the PFM is necessary for the understanding of women over their own bodies, facilitating the understanding of the guidelines and treatments offered by health professionals. Communication and information are essential in the treatment of patients with PFDs. The correct information is important in obtaining the patient's consent on the proposed therapy in treatment, increasing their participation, reducing anxiety, providing knowledge about the disease and assessing the patients' satisfaction with the results.

## Notes

Appendix 1 Search strategy on PubMed ((“knowledge”[MeSH Terms] OR “knowledge”[All Fields]) OR (“comprehension”[MeSH Terms] OR “comprehension”[All Fields]) OR (“education”[Subheading] OR “education”[All Fields] OR “educational status”[MeSH Terms] OR (“educational”[All Fields] AND “status”[All Fields]) OR “educational status”[All Fields] OR “education”[All Fields] OR “education”[MeSH Terms]) OR “education level”[All Fields]) AND (“urinary incontinence”[All Fields] OR “pelvic organ prolapse”[All Fields] OR “genital prolapse”[All Fields] OR “stress urinary incontinence”[All Fields] OR (urgence[All Fields] AND (“urinary incontinence”[MeSH Terms] OR (“urinary”[All Fields] AND “incontinence”[All Fields]) OR “urinary incontinence”[All Fields])) OR “mixed urinary incontinence”[All Fields] OR (“cystocoele”[All Fields] OR “cystocele”[MeSH Terms] OR “cystocele”[All Fields]) OR (“rectocoele”[All Fields] OR “rectocele”[MeSH Terms] OR “rectocele”[All Fields]) OR “apical prolapse”[All Fields] OR “uterine prolapse”[All Fields] OR “overactive bladder”[All Fields] OR “detrusor overactivity”[All Fields]) NOT (m?n[All Fields] OR (animal[All Fields] OR animal'[All Fields] OR animal'naia[All Fields] OR animal'no[All Fields] OR animal's[All Fields] OR animal1[All Fields] OR animal2[All Fields] OR animal80[All Fields] OR animala[All Fields] OR animalae[All Fields] OR animaland[All Fields] OR animalarial[All Fields] OR animalario[All Fields] OR animalarium[All Fields] OR animalas[All Fields] OR animalbase[All Fields] OR animalbehavior[All Fields] OR animalbehaviorclinic[All Fields] OR animalbehavior[All Fields] OR animalbiology[All Fields] OR animalcare[All Fields] OR animalcentric[All Fields] OR animalcentro[All Fields] OR animalci[All Fields] OR animalclinicofregina[All Fields] OR animalcognition[All Fields] OR animalcompassionfoundation[All Fields] OR animalconcepts[All Fields] OR animalconsultants[All Fields] OR animalcula[All Fields] OR animalcular[All Fields] OR animalcule[All Fields] OR animalcules[All Fields] OR animalcules'[All Fields] OR animalculi[All Fields] OR animalculi'[All Fields] OR animalculis[All Fields] OR animalculos[All Fields] OR animald[All Fields] OR animaldairy[All Fields] OR animalde[All Fields] OR animaldentalcenter[All Fields] OR animaldentalservices[All Fields] OR animaldepartamento[All Fields] OR animalderived[All Fields] OR animaldocfox[All Fields] OR animale[All Fields] OR animale'[All Fields] OR animalearn[All Fields] OR animaleau[All Fields] OR animaledu[All Fields] OR animalefaculte[All Fields] OR animalele[All Fields] OR animalelimogesfrance[All Fields] OR animalelor[All Fields] OR animalem[All Fields] OR animalemergency[All Fields] OR animalen[All Fields] OR animalendocrine[All Fields] OR animaleolor[All Fields] OR animaler[All Fields] OR animalerc[All Fields] OR animalerie[All Fields] OR animaleries[All Fields] OR animales[All Fields] OR animales'[All Fields] OR animalessin[All Fields] OR animaletoulouse[All Fields] OR animaletti[All Fields] OR animaleuniversite[All Fields] OR animalexp[All Fields] OR animaleyecare[All Fields] OR animaleyedoctor[All Fields] OR animalfacultad[All Fields] OR animalfeed[All Fields] OR animalfeeds[All Fields] OR animalfree[All Fields] OR animalgenome[All Fields] OR animalgesic[All Fields] OR animalgrupo[All Fields] OR animalh[All Fields] OR animalhealth[All Fields] OR animalhealthaustralia[All Fields] OR animalhealthireland[All Fields] OR animali[All Fields] OR animalia[All Fields] OR animalian[All Fields] OR animalibus[All Fields] OR animalibus'[All Fields] OR animalic[All Fields] OR animalien[All Fields] OR animalier[All Fields] OR animaliere[All Fields] OR animalieres[All Fields] OR animalies[All Fields] OR animaliieiu[All Fields] OR animalin[All Fields] OR animalinia[All Fields] OR animalinla[All Fields] OR animalinsides[All Fields] OR animalinstituto[All Fields] OR animalis[All Fields] OR animalisante[All Fields] OR animalisation[All Fields] OR animalische[All Fields] OR animalischem[All Fields] OR animalischen[All Fields] OR animalischer[All Fields] OR animalises[All Fields] OR animalisierte[All Fields] OR animalism[All Fields] OR animalisssp[All Fields] OR animalistic[All Fields] OR animalistol[All Fields] OR animalists[All Fields] OR animalite[All Fields] OR animality[All Fields] OR animalium[All Fields] OR animalium'[All Fields] OR animaliumque[All Fields] OR animalivore[All Fields] OR animalivores[All Fields] OR animalivorous[All Fields] OR animalization[All Fields] OR animalizatsii[All Fields] OR animalize[All Fields] OR animalized[All Fields] OR animalizes[All Fields] OR animalizing[All Fields] OR animalizing'[All Fields] OR animalizzante[All Fields] OR animalkulismus[All Fields] OR animall[All Fields] OR animallectindb[All Fields] OR animallectindb'[All Fields] OR animallee[All Fields] OR animallevel[All Fields] OR animallifesolutions[All Fields] OR animalloimia[All Fields] OR animalls[All Fields] OR animally[All Fields] OR animalmedical[All Fields] OR animalmedicine[All Fields] OR animalmodel[All Fields] OR animalmodels[All Fields] OR animalmost[All Fields] OR animalne[All Fields] OR animalneho[All Fields] OR animalness[All Fields] OR animalni[All Fields] OR animalnich[All Fields] OR animalnih[All Fields] OR animalniho[All Fields] OR animalnim[All Fields] OR animalnl[All Fields] OR animalnu[All Fields] OR animalny[All Fields] OR animalnych[All Fields] OR animaloid[All Fields] OR animalomics[All Fields] OR animaloris[All Fields] OR animalous[All Fields] OR animalpathogene[All Fields] OR animalpathogenic[All Fields] OR animalphysio[All Fields] OR animalproductivity[All Fields] OR animalqtldb[All Fields] OR animalrelated[All Fields] OR animalreproduction[All Fields] OR animals[All Fields] OR animals'[All Fields] OR animals'behavior[All Fields] OR animals'behavioral[All Fields] OR animals'equally[All Fields] OR animals'fibrinolytic[All Fields] OR animals'hepatic[All Fields] OR animals'hips[All Fields] OR animals'life[All Fields] OR animals'motor[All Fields] OR animals's[All Fields] OR animals'serum[All Fields] OR animals'skin[All Fields] OR animals'suboriferous[All Fields] OR animals2[All Fields] OR animals2,3[All Fields] OR animals37[All Fields] OR animalsa1[All Fields] OR animalsand[All Fields] OR animalsaustralia[All Fields] OR animalscan[All Fields] OR animalschangchun[All Fields] OR animalscience[All Fields] OR animalsciences[All Fields] OR animalscommunities[All Fields] OR animalsdepartment[All Fields] OR animalses[All Fields] OR animalsethical[All Fields] OR animalsha[All Fields] OR animalsin[All Fields] OR animalsinstitute[All Fields] OR animalsk[All Fields] OR animalskawasaki[All Fields] OR animalskawasakijapan[All Fields] OR animalske[All Fields] OR animalskinallergy[All Fields] OR animalsmin[All Fields] OR animalspecialtycenter[All Fields] OR animalspecies[All Fields] OR animalsperformance[All Fields] OR animalsr[All Fields] OR animalsroyal[All Fields] OR animalss[All Fields] OR animalsschool[All Fields] OR animalssix[All Fields] OR animalssun[All Fields] OR animalsthe[All Fields] OR animalsthis[All Fields] OR animalstreated[All Fields] OR animalsu[All Fields] OR animalsunder[All Fields] OR animalsversus[All Fields] OR animalswere[All Fields] OR animalswild[All Fields] OR animaltfdb[All Fields] OR animalthe[All Fields] OR animaltissues[All Fields] OR animaltracker[All Fields] OR animalu[All Fields] OR animalul[All Fields] OR animalultrasound[All Fields] OR animalului[All Fields] OR animaluniversidade[All Fields] OR animalvegetative[All Fields] OR animalviren[All Fields] OR animalward[All Fields] OR animalwards[All Fields] OR animalwelfare[All Fields] OR animalwere[All Fields] OR animalwith[All Fields] OR animalx[All Fields] OR animalx100[All Fields] OR animalxside[All Fields] OR animaly[All Fields] OR animalytics[All Fields] OR animalzellen[All Fields]))
